# Nurses’ perceptions of an audiovisual material to support patient education on oral health management in diabetes care

**DOI:** 10.1371/journal.pone.0361280

**Published:** 2026-10-21

**Authors:** Rie Kudoh, Taiga Shibayama

**Affiliations:** Institute of Medicine, University of Tsukuba, Tsukuba, Ibaraki, Japan; University of the Witwatersrand Johannesburg Faculty of Health Sciences, SOUTH AFRICA

## Abstract

Educational support for nurses is essential to promote oral health management education for patients with diabetes. However, few studies have focused on supporting nurses in this context. This study explored the effects of an audiovisual material designed to support nurses’ patient education on oral health management in diabetes care. Semi-structured interviews were conducted with eight certified diabetes nurse educators. Data were analyzed using content analysis. Four main themes emerged: (1) enhancement of nurses’ individual learning (8/8 participants), including improved knowledge of the connection between oral health and diabetes; (2) promotion of professional development and reflection (5/8); (3) provision of new insights into patient care (7/8), particularly regarding patient motivation strategies; and (4) identification of implementation considerations in diabetes outpatient settings. The findings suggest that the audiovisual material, designed to complement an existing evidence-based educational guide, was perceived as useful and may support nurses’ learning and educational practices related to oral health education for patients with diabetes.

## Introduction

Periodontal diseases are chronic inflammatory conditions caused by microbial dysbiosis and an impaired host response, affecting the periodontium [1]. This host response can be influenced by systemic conditions. In particular, poorly controlled diabetes markedly increases the risk, severity, and progression of periodontitis through mechanisms involving oral microbial dysbiosis, chronic inflammation, immune dysregulation, and altered bone metabolism [2]. Disease progression can lead to destruction of the periodontium and tooth loss [3]. In 2021, severe periodontal disease affected over 1 billion people globally, and edentulism affected approximately 353 million, and these numbers are expected to continue increasing [4]. Given the rapidly aging population in Japan, this global trend is particularly relevant in the Japanese context. A recent report found that 40.0% of people aged 45–54 had at least one missing tooth, with the rate rising to 93.5% among those aged 75 and older [5], indicating that tooth loss is highly prevalent among middle-aged and older adults in Japan.

Evidence of an interrelationship between diabetes and periodontal disease has been reported in many reviews and epidemiological studies [6,7]. Diabetes mellitus represents a risk factor for developing and worsening periodontal disease, with the risk of progression increasing approximately threefold in diabetic patients compared with non-diabetic patients [8]. The 2017 classification of periodontal and peri-implant diseases and conditions recognizes diabetes, particularly when poorly controlled, as a risk factor for the progression of periodontitis [9]. Periodontal disease can cause tooth loss, impaired mastication, nutritional deficiencies, and reduced quality of life [10]. It can also lead to poor glycemic control and may affect the onset and progression of diabetes complications, such as retinopathy, neuropathy, nephropathy, or cardiovascular disease [11]. Therefore, early detection of periodontal disease from both dental and medical perspectives is essential for effective management and prevention. Effective oral health management in diabetes care has been shown to contribute to improved glycemic control and reduced risk of complications, ultimately enhancing patients’ overall health outcomes and quality of life [11].

Previous studies have shown that many patients with diabetes have insufficient awareness and knowledge of oral health management and rarely consult dental professionals unless symptoms such as gingival bleeding, gingival swelling, pain, or tooth mobility appear [12]. This highlights the need for systematic oral health education as part of diabetes self-management.

Certified diabetes nurse educators (CDNEs), who are nurses with advanced and broad expertise in providing self-management education to outpatients with diabetes, can play a vital role in the oral health management of outpatients with diabetes [13]. Given the inadequate oral care practices among diabetic patients and the strong bidirectional relationship between diabetes and periodontal disease [6–8,10,11], there is a critical need for CDNEs to provide comprehensive oral health education as an integral component of diabetes self-management education. CDNEs are uniquely positioned to reach patients during regular diabetes care visits, providing opportunities for repeated reinforcement of oral health messages and bridging the gap between medical and dental care [13]. Previous research has indicated that CDNEs face several barriers to providing oral health education, including limited training opportunities, uncertainty about appropriate teaching content, and a lack of interprofessional collaboration with dental professionals [12]. These barriers are multifaceted: many CDNEs report that oral health is not adequately covered in their professional training programs, leaving them uncertain about which oral assessment techniques are appropriate for their scope of practice and how to effectively communicate oral health information to patients [12]. The absence of standardized protocols and clear guidance on interprofessional collaboration further compounds these challenges, resulting in inconsistent delivery of oral health education across diabetes care settings [12,14]. As a result, nurses may feel unprepared or lack confidence in delivering oral health education effectively. To address this gap, the authors previously developed a nursing guide for oral health management as a self-learning tool that can be used in diabetes outpatient clinics [14]. The guide was evaluated positively, helping to increase nurses’ knowledge and confidence in oral assessment and promoting oral health management in patients with type 2 diabetes [14,15]. While the nursing guide has proven effective in enhancing nurses’ knowledge and confidence [14,15], feedback from users indicated that written materials alone have limitations in conveying practical skills and techniques. Specifically, nurses expressed the need for visual demonstrations of oral assessment procedures, patient communication strategies, and step-by-step guidance for conducting oral health education sessions in clinical practice [15]. These findings indicated a need for supplementary video-based audiovisual materials, in addition to the existing guide, to support visual learning for practical implementation in clinical settings. Audiovisual materials can provide dynamic demonstrations that are difficult to convey through text and static images alone, allowing learners to observe realistic patient interactions and assessment techniques. Such materials also accommodate diverse learning styles and can be accessed repeatedly for skill reinforcement [15]. Therefore, the authors developed an audiovisual material to complement the existing nursing guide [14] and to support nurses in delivering patient education on oral health management in diabetes care.

Despite the recognized importance of nurses’ role in oral health education for diabetes care, there is limited research examining effective educational interventions to enhance nurses’ capacity in this area. While previous studies have identified barriers faced by nurses [12], few have evaluated educational programs designed to address these barriers or explored nurses’ learning experiences with such interventions [14,15]. Furthermore, the potential of audiovisual educational materials in supporting nurses’ learning about oral health management in diabetes care remains largely unexplored.

This study aimed to explore the effects of audiovisual material, developed to support nurses’ learning about oral health management in diabetes care, on nurses’ patient education practices.

## Materials and methods

### Study design

This study adopted a qualitative design to explore nurses’ subjective learning experiences, perceptions, and reflections after using the audiovisual material. This approach was appropriate for capturing in-depth insights into how the material influenced nurses’ understanding and practice regarding oral health management in diabetes care. In this study, the term “effect” was operationally defined as perceived changes in nurses’ knowledge, attitudes, and practices related to oral health management, rather than direct patient outcomes. These changes in nurses are expected to ultimately contribute to improved patient care.

The study flowchart is presented in Fig 1.

**Fig 1:**
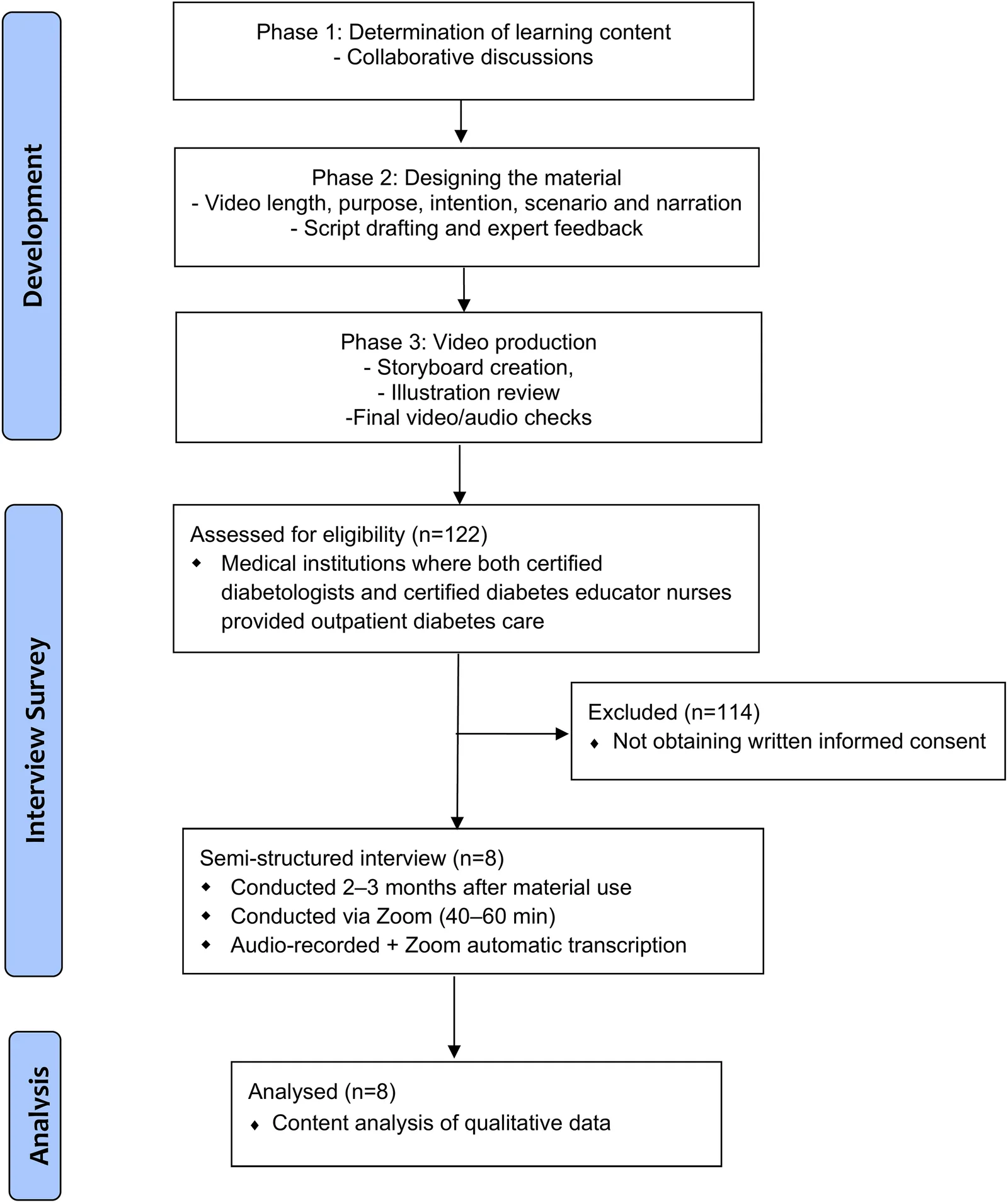
Study flowchart showing the development process and evaluation of the audiovisual material. The audiovisual material was developed through a three-phase process: Phase 1, determination of learning content through collaborative discussions with certified diabetologists and certified diabetes educator nurses; Phase 2, designing the material, including video length, purpose, intention, scenario, and narration, followed by script drafting and expert feedback; and Phase 3, video production, including storyboard creation, illustration review, and final video/audio checks. For the feasibility evaluation, institutions where both certified diabetologists and certified diabetes educator nurses provided outpatient diabetes care were assessed for eligibility (n = 122); 114 were excluded because written informed consent was not obtained. The remaining 8 nurses underwent a semi-structured interview conducted via Zoom (40–60 min) 2–3 months after using the material; interviews were audio-recorded and automatically transcribed, and the transcripts were analyzed (n = 8) using qualitative content analysis.

In this study, “oral health management” was defined as a comprehensive approach that includes observation and assessment of patients’ oral cavities; patient education related to oral health management, such as maintaining oral hygiene, ensuring adequate oral moisture, and denture care; and documentation to support the determination of an appropriate course of action [16].

The audiovisual material was developed in three phases: determining the learning content, designing the material, and producing the video.

1. The learning content was determined through collaborative discussions among nursing researchers specializing in diabetes care and oral health management, as well as dentists and dental hygienists. The material was designed to help nurses, particularly those interested in providing patient education on oral health management in diabetes care, understand key aspects of the relationship between periodontal disease and diabetes. Specifically, the material conveys that periodontal disease is a complication associated with poorly controlled diabetes, and that its progression can be prevented through appropriate self-care of both periodontal disease and type 2 diabetes as part of a comprehensive management program [17]. It also emphasizes the importance of regular dental visits and highlights that patient education on oral health management plays an important role in preventing diabetes-related complications. The content enables nurses to grasp key points of oral health support by reviewing actual patient education situations.
2. The design of the audiovisual material was examined through discussions between diabetes nursing and oral health management researchers regarding video length, purpose, scenario, and narration elements. These aspects and source materials were discussed repeatedly between the researchers and a professional video production company. The first draft of the script was then evaluated by dentists and nurses involved in outpatient diabetes care to ensure content validity, practical relevance, and clarity, and their feedback was incorporated into the final version.
3. Video production was outsourced to a specialized company and carried out in three stages: storyboard creation, illustration review, and final video/audio checks. At each stage, the content was refined through discussions between the production company and researchers. Through this iterative process, the audiovisual material was completed.

### Content of the audiovisual material

The audiovisual material was designed to achieve the following learning objectives for nurses: (i) to enable nurses to visualize the process of observing and assessing the oral cavity of patients with diabetes in an outpatient diabetes department, and (ii) to enable nurses to perform oral cavity observation and assessment for patients with diabetes in an outpatient setting, focusing on the specific assessment elements described below (see Key Assessment Points).

1 Duration: The video was approximately 15 minutes long.
2 Visual aids: The video used animation to explain the content and included oral cavity photographs provided by dentists and by a local dental hygienists’ association (an organization of dental hygienists in the region), with appropriate permissions obtained for their use.
3 Specific scenarios: The video consisted of four sections: (i) Introduction, (ii) Key Assessment Points, (iii) Teaching moments, and (iv) Ending.
  (i) Introduction: Explained the importance of oral health education provided by nurses in diabetes care and included a true/false quiz format to help learners identify correct oral health management practices.
  (ii) Key Assessment Points: Explained how to assess overall oral condition, oral hygiene, dry mouth, halitosis, subjective symptoms of periodontal disease, and the number of remaining teeth, using actual oral cavity photographs.
  (iii) Teaching moments: Visually presented the sequence of a nurse–patient interaction in an outpatient diabetes department, allowing learners to grasp both the practical process of oral cavity observation and assessment, and the appropriate types of communication to use with patients.
  (iv) Ending: Highlighted the current state of oral health management in diabetes care, the importance of medical–dental collaboration, and delivered an encouraging message to build nurses’ confidence in implementing oral health management.
4 Narration: Each section was accompanied by narration to guide viewers through the content.

### Participants

Participants were recruited from among CDNEs engaged in diabetes care in outpatient departments in three prefectures of the Kanto region of Japan. We identified 122 medical institutions where both certified diabetologists and CDNEs provided outpatient diabetes care by matching data from the Japan Diabetes Society database and the Certification Board for Diabetes Educators in Japan database [18,19]. A purposive sampling approach was used to select CDNEs who were involved in diabetes outpatient care. As the number of CDNEs at each institution was unknown, we mailed a study invitation to a CDNE involved in outpatient diabetes care at each institution. After obtaining written informed consent from the CDNE on behalf of the institution, the researcher mailed the QR code of the audiovisual material to the institution between November 2024 and January 2025. The inclusion criterion was current employment as a CDNE at the institution at the time of the interview. CDNEs without experience in diabetes outpatient settings or those who declined participation were excluded.

### Data collection

The interviews were conducted by the first author using a semi-structured interview guide to ensure consistency in data collection and enhance the reliability of the study. This interview guide was designed to qualitatively assess the effectiveness of the audiovisual material, examining how the learning objectives were reflected in nurses’ knowledge, attitudes, and practices. The interview guide was developed based on the study objectives and previous literature on diabetes and oral health education. Written informed consent was obtained from all participants prior to study participation. All interviews were conducted via Zoom [20], audio-recorded, and transcribed using Zoom’s automatic transcription feature. Prior to the start of each interview, the researcher explained the purpose of audio recording, how the recordings would be stored and used, and participants’ right to decline recording or to withdraw at any time. Verbal consent to be audio-recorded was then obtained from each participant and recorded at the beginning of the interview, before the substantive interview questions began. Transcripts were manually reviewed to ensure accuracy. Participants were also asked to complete a survey, which allowed the research team to collect demographic data prior to the interview. Participation in the study was voluntary, and no compensation was provided. After the QR code for the audiovisual material was mailed, the researcher contacted the participants to arrange a time for an interview, 2–3 months after the audiovisual material was used in patient education. Interview times were arranged based on mutual availability. All interviews were conducted in a quiet setting that ensured participant privacy. Each interview lasted between 40 and 60 minutes. To ensure content validity, the interview guide was reviewed by two researchers: one with expertise in diabetes nursing and another with expertise in the oral health management of diabetes, both affiliated with the same institution.

The interview guide and face sheet included the following sections:

### Interview guide

1. Why did you decide to use the audiovisual material?
2. How did you apply what you learned from the audiovisual material to the observation and assessment of oral health in patients with diabetes?
3. How did what you learned from the audiovisual material help you to observe and assess patients’ oral cavities?
4. How did your patients react when you performed the oral observations and assessments that you learned about through the audiovisual material?
5. What have you noticed from observing and assessing patients’ oral cavities regarding the oral health management you perform and its effects on the surrounding medical staff?
6. To what extent do you think nurses can provide oral health management support in diabetes outpatient departments?
7. In addition to the content of the audiovisual material, what innovations do you think will help nurses and patients accept the ongoing practice of oral health management in diabetes care settings?
8. What would you like to learn in addition to what was covered in this audiovisual material?

### Face sheet of participants’ demographics

Participants’ demographic information included gender, age, work experience after becoming a CDNE, and the type of work institution. Additionally, information regarding education on the oral health management of patients with diabetes, and the existence of a dentistry department at their institution was collected using a dichotomous scale (1 = yes, 0 = no).

### Data analysis

Qualitative data were analyzed using content analysis, which involves classifying words and phrases from textual data into categories based on recurring ideas or thought patterns [21,22]. Verbatim transcripts were created from the audio recordings and read repeatedly to gain familiarity with the content. The first author, a nursing researcher with expertise in oral health management in diabetes, identified and coded key phrases and sentences relevant to the research questions, and grouped emerging codes into subcategories, categories, and themes based on conceptual similarity and relevance to the study objectives. A second researcher specializing in diabetes care independently reviewed and re-coded portions of the transcripts, and discrepancies were resolved through discussion until consensus was reached on the final themes, categories, and subcategories. MAXQDA software (VERBI GmbH) [23] was used to organize, code, and retrieve data throughout the analysis.

### Trustworthiness

To ensure the trustworthiness of the qualitative findings, several strategies were employed. Coding and analysis were independently conducted by two researchers, who cross-checked their coding to enhance the credibility and confirmability of the analysis. Discrepancies in coding were resolved through discussion and peer debriefing within the research team until consensus was reached. The interview guide and analytical framework were developed and refined through discussion between the two researchers prior to data collection. Member checking with participants was not performed; therefore, the interpretations presented reflect the researchers’ analysis of the interview data rather than participant-confirmed accounts.

### Bracketing and reflexivity

The research team’s professional backgrounds and perspectives were considered in relation to their potential influence on the study. RK is a nurse and researcher with expertise in oral health management and diabetes nursing, and TS is a nurse and researcher with expertise in diabetes nursing. RK’s clinical experience and interest in oral health management informed the motivation for this study. Neither researcher had any prior personal or professional relationship with the participants, which reduced the likelihood of relational bias during data collection. To minimize the influence of the researchers’ backgrounds and prior assumptions on data interpretation, the interview guide was used consistently across interviews, coding was conducted independently by two researchers, and discrepancies were resolved through discussion and peer debriefing, as described above. Through this process, the researchers sought to bracket their own clinical experiences and assumptions and to remain open to participants’ perspectives as expressed in the interview data.

### Ethical considerations

This study was approved by the ethics committee of the authors’ affiliated university (approval number: 2039; approval date: 01/10/2024).

## Results and discussion

### Results

Of the 122 CDNEs initially recruited by the researcher, eight provided written informed consent and participated in this study, and data from those participants were used in the analysis. Verbal consent was also obtained at the beginning of each interview to reconfirm participants’ willingness to participate and to be audio-recorded. The participants’ demographic characteristics are presented in Table 1.

**Table 1:** Participants’ demographic characteristics.

	A	B	C	D	E	F	G	H
Gender	Male	Female	Female	Female	Female	Female	Female	Female
Age	30s	40s	40s	30s	40s	60s	40s	40s
Work experience as a nurse (years)	13	22	28	10	17	37	25	25
Work experience as a CDNE^†^ (years)	4	7	10	5	5	15	20	20
Experience in oral health management education of patients with diabetes	No	No	Yes	No	No	Yes	Yes	Yes
Work institution type	Hospital	Hospital	Hospital	Clinic	Hospital	Hospital	Hospital	Hospital
Existence of a dentistry department at the work institution	No	Yes	No	Yes	Yes	Yes	Yes	No

†Note: CDNE: certified diabetes nurse educator.

Letters A–H were used to label the participants.

Participants ranged in age from their 30s to their 60s, with over half in their 40s. The participants’ work experience as CDNEs ranged from 4 to 20 years, and half of them had received education on oral health management.

Four overarching themes and eleven categories were generated, representing participants’ reflections and learning after engaging with the audiovisual material. While Themes 1 and 2 reflect nurses’ direct learning experiences related to the audiovisual material, Themes 3 and 4 represent broader, contextual reflections that emerged as participants considered the application of the material within their clinical practice. These latter themes are presented to provide contextual understanding rather than to indicate direct effects of the audiovisual material. To enhance clarity, the main findings for each theme are summarized below, and detailed supporting quotes are presented in Table 2.

**Table 2:** Nurses’ feedback after watching the audiovisual material and selected participant quotes.

Category	Subcategory	Participants, n	Example quotes
**Theme 1: Individual Learning Enhancement**			
Helped to understand how to approach patients and observe their oral cavity. (n = 8)	Specific ways to approach patients and observe their oral cavity are easier to visualize than in writing.	8	It was easier to understand how to approach patients because the information was not only presented in text, but also visually and audibly, with supporting images. (G5)
			Scenes such as observing patients through a mirror were easier to understand than reading about them in text, and useful for patient education. (B82)
			Seeing real examples of what not to say and how not to ask things made it really easy to understand and helpful. (F84)
	Pictures and expressions can be used directly for patient education.	4	The photos in the audiovisual material could be shared with patients and used to encourage them to visit a dentist. (E197)
			The audiovisual material was clearly expressed, so I felt it could be shown directly to patients and still be easily understood. (C208)
	The goals of oral health management are clearer.	2	The goals of oral health management are clearer. (A426)
	The time spent watching the video was an appropriate length.	2	It was easy to find time because the video was on YouTube, and I knew it was about 10 minutes long. The length of time is also very good. (G5)
			It would be better if it were a little shorter—about five minutes—so it’s easier to watch while waiting. (H38)
	Using the videos together with the guide helps deepen understanding.	2	The content of the audiovisual material was linked to the guide, so it was easy for me to understand. Being familiar with the guide helped me explain the need for oral health management to patients. (C2)
Helped to check patients’ oral symptoms, observe oral conditions and provide patient education in practice. (n = 8)	Able to educate the patient on oral self-management.	5	I provided education based on the audiovisual material, which served as a reference. (A146)
			In addition to encouraging dental visits, I was able to talk about related issues such as gingival bleeding during brushing, and oral hygiene habits. (E182)
			When patients mentioned having difficulty eating due to dental problems, I began to take a more proactive approach by asking about their dental visit history and their oral condition. (G38)
	Observed patients’ oral cavity.	4	I asked patients to self-check their oral cavities. When I called to them from behind at an angle, they adjusted their view to observe their oral cavity from different perspectives. (D72)
	Able to assess oral symptoms.	1	It was difficult to assess which oral symptoms warranted a dental visit, but it was possible to classify the severity of the condition as either severe or mild. (D405)
**Theme 2: Professional Development and Reflection**			
Motivated me and other staff to practice oral health management. (n = 5)	Led to a common understanding with surrounding staff	3	The use of audiovisual materials allowed for a common understanding of patient education for us. (C104)
	Motivated me and staff	3	This kind of opportunity made me think a lot about how I should intervene with patients. (A470)
			I believe the audiovisual material is sufficiently motivating for both the staff and myself. (B209)
	Gave an opportunity to reflect on my own oral health management.	2	I realized that I had not been practicing oral health management, even though it was necessary. (B176)
	Led to practical oral health management by staff.	1	Other staff have told me they confirm the status of dental visits using the dentistry section of the cooperation handbook. (C135)
	Enhanced self-confidence.	1	Having a pathway to follow—such as audiovisual materials—helps me feel more confident when teaching. (A115)
	Information not shared with other staff.	5	I was the only one who practiced oral health management. (H34)
Provided an opportunity to reflect on the oral health management I have provided to patients thus far. (n = 6)	Rarely implemented oral health management practices.	4	I rarely implemented oral health management practices, and the other staff hardly do it for patients with diabetes. (A140)
			We have not included oral health topics in diabetes classes so far, and it has rarely been addressed in outpatient education. (D195)
	Checking dental visit status	2	Dental visit status is checked in the coordination handbook. (C70)
	Recommendation for dental consultation	2	Patients completed the oral health self-check items, and based on the results, a dental visit was recommended. (D288)
	Education is provided exclusively to patients presenting with symptoms of concern.	1	Oral health management is given at the end of the diabetic complications topic, but only to patients showing concerning oral symptoms. (D92)
Gave an opportunity to reflect on the state of medical-dental collaboration. (n = 5)	There is no collaboration with dentistry.	3	There is no collaboration with dentistry regarding the care of patients with diabetes within the hospital. (B149)
	Consultations with dental professionals do occur occasionally, although not routinely.	3	I occasionally talk to dentists in the hallway if something’s on my mind about patients’ oral condition. (G107)
			When patients with diabetes are going into surgery, I sometimes ask dental staff to explain oral health issues, considering their diabetes. (C52)
	Willingness to collaborate with dentistry.	3	I would like to collaborate with the dental team. It would really help if there were dental hygienists who could care for patients with diabetes. (G137)
**Theme 3: Patient Care Insights**			
Gained insights into patients’ perceptions of oral health management. (n = 7)	Low interest in oral health.	6	Whatever I said, their reaction was rather muted—just ‘hmm,’ not much of a response. (A337)
			Patients in their 40s and 50s—so, relatively young—often don’t pay attention to the need to visit a dentist. Maybe it’s because, for them, keeping up with work and daily life is the top priority. (C156)
			Many patients didn’t really go to the dentist. When I asked, they said it’s because it doesn’t hurt much, so they don’t go—even if they have cavities. (H51)
	Need to be considerate when asking questions about oral health.	4	A few years ago, I asked a patient with a ragged tooth if she went to the dentist. She seemed really shocked. I realized that oral health can be a sensitive and private topic. (B98)
	Discomfort with oral cavity observation.	4	Some patients were reluctant to let us look inside their oral cavity. They seemed to feel a sense of shame. (D14)
			Those with dentures were especially likely to refuse. (C33)
	Lack of awareness about diabetes-periodontal disease relationships.	4	When I talked to patients, many of them didn’t realize there is a link between oral health and diabetes. (H54)
	Patients taking care in their own way.	1	Some said, ‘I’m brushing my teeth because otherwise I won’t be able to enjoy good food.’ (A339)
	No resistance to being asked about oral health	1	When I looked at their diabetes notebook and asked, ‘Can I take a look in your mouth?’ or ‘Have you been to the dentist?’, most patients didn’t mind answering. (C30)
	No resistance to having the oral cavity observed.	2	When I spoke to patients I’d been seeing for foot care over a long time, they opened their mouths and showed their oral cavity without much hesitation. (H64)
Gained insights into patients’ actual oral health conditions. (n = 5)	Had a very unclean oral cavity.	4	The patient had not brushed, so there was bad breath, a lot of food debris and tartar. But I wasn’t surprised because I expected it to look like this when I saw the patients’ feet. (A305)
			Self-care is about paying attention to your own body. Patients who don’t care about their oral health or their feet are not really concerned about their health. (H71)
			There was definitely some bad breath. The patient had a partial denture, and it seemed like it had probably been left in for a long time. (B66)
	Tooth loss caused difficulties with eating.	4	Some people said they just left it as it was because it didn’t affect them, even though they had no teeth and were in a terrible condition. (D138)
			The person in the worst condition had no upper teeth when I looked inside the oral cavity. The bridge had broken, so they couldn’t wear dentures, and they were just grinding food with their lower teeth and upper jaw before swallowing. But that seemed normal for them. (D165)
	There are no major problems in the oral cavity.	1	The patient is elderly and does not attend regular dental check-ups. There were no abnormalities in the gums. I had the impression that they were relatively clean and regularly brushed. (A329)
**Theme 4: Implementation Considerations**			
Scope of oral health management that nurses can perform in the diabetes outpatient department. (n = 7)	Oral health management instruction.	6	I think it would be better to provide education on toothbrushing techniques and also try plaque disclosure. (C405)
	Oral cavity observation	5	It is possible to observe the patients’ oral cavity. (H87)
	Providing information to patients	4	As staff involved in diabetes care, I believe it’s very important to inform patients about its impact on overall health. (C313)
	Motivating patients toward oral health	3	I think it would be good if we could motivate patients [toward oral health management]. (E117)
	Assessing oral condition and encouraging dental visits	3	By simply following the methods in the audiovisual material, anyone can assess a patient’s oral condition and encourage a dental visit. (D259)
	Check the oral symptoms	2	Since we talk one-on-one, it’s possible to check for halitosis. But I worry that mentioning halitosis might make the patient feel embarrassed or shut down. (E96)
			It is good if patients could self-check their oral cavity and nurses could assess the results. (G65)
Additional learning needs beyond the current content of the material. (n = 5)	Request for more images of diverse oral conditions.	5	I want to see more pictures of various oral conditions. It would be easier to understand if there were pictures of the same person in a healthy and unhealthy oral condition. (G193)
	Examples of oral health management support in other facilities.	4	I would like to know more examples of the kind of support provided to patients in other facilities, as it may be useful for practice in my facility. (A419)
			I want to know about facilities that practiced oral health management, such as how the patients are doing, what they actually struggled with, what was good about it, and how to start. (C450)
	Practical consolidation of audiovisual learning.	2	Because it has just started, I want to first consolidate what I have learned from the audiovisual material in practice. (B211)
Ingenuity to help nurses and patients accept oral health management. (n = 8)	Ingenuity to reduce patients’ shame about having their oral cavity observed.	8	If we had a diabetes care room with a water supply and space for toothbrushing, we could observe [patients’ oral cavity]. However, the lack of such facilities makes it more difficult. (G67)
			The patients I worked with were highly resistant. Consideration needs to be given to how to reduce the resistance to opening their oral cavities. (C273)
			Asking patients to show their oral cavity at the first visit might catch them off guard. It would be more considerate to have them show it at the next visit, because they probably want to brush their teeth well before doing so. (A367)
	Need a trigger to observe patients’ oral cavity.	6	It could be possible to implement if there is some kind of trigger to observe patients’ oral cavities. (B110)
			Through discussions about dietary habits—such as difficulty eating hard foods—some individuals reveal that they either do not have dentures or choose not to use them due to poor fit. (F112)
	Need for educational materials that can help explain to patients including self-checks by patients.	3	Using a booklet would make the explanation easier. If a booklet version of the audiovisual material is available, I can give it to patients and indicate which part to pay attention to. (E118)
			It would be good if there were tools for patients to self-check at home to raise awareness. (B203)
	Audiovisual materials for patients.	3	Even if nurses are motivated, observing patients’ oral cavities is difficult unless the patients themselves are interested. So, it would be better for patients to watch [an audiovisual material] designed for patients. (G197)
	Additional medical reimbursement.	2	It should be eligible for additional medical reimbursement. If this is applied, it can serve as motivation, as it would become part of formal work. (A381)
	Need various ways to learn about oral health management.	2	Stimulation is necessary, for example through training courses. This requires a learning environment such as hospital training, study groups, or YouTube. (A399)
	Need to repeat the message	1	If we set aside a period every year—like an “oral health management and Diabetes Week”—and keep doing it for years, it’ll probably start to feel normal. I feel like the only way is to just keep repeating the message. (H78).
	Implement during educational hospitalization.	1	We usually don’t have time to talk about oral health management in the outpatient setting, but I thought it might be easier to explain if it were included as part of inpatient education. (D190)
	Need to take into account patients’ financial issues.	1	When I asked, “Do you not go to the dentist or have dentures made?”, the patients mentioned financial difficulties. So, I couldn’t suggest making dentures. (F113)
		1	I think patients feel nervous when a nurse stands behind them as they look at their oral cavity in the mirror. The level of embarrassment varies, but it seems less awkward when the nurse is focused on the computer. (A154)
	A telenursing approach.	1	If it were done through telenursing rather than in person, patients might be willing to let us observe their oral cavity. (G163)
	An approach using an oral cavity model to educate patients.	1	Just as dentists use tooth models, it would be good to have such a model in patient education related to oral health. For example, patients can use the model to point to where it hurts or where a cavity is. This may provide an opportunity for nurses to observe the patients’ oral cavities. (C273)
Issues to overcome in future clinical practice. (n = 7)	Lack of time to provide oral health education to patients.	7	Given the limited time available for each individual patient, I don’t have enough time to observe their oral cavity. (G70)
	Lack of interest of other staff.	5	I want other staff to be more interested and learn how to approach patients. (A230)
	Observation of patients’ oral cavity is difficult.	4	Even when I ask patients to open their mouths, few respond willingly—even those who visit the dentist regularly. (F94)
	Low confidence in the ability to assess oral condition.	2	I haven’t been trained to assess periodontal disease, so I lack confidence and feel hesitant to examine patients’ oral cavity. (G54)
	Did not lead to continuous follow-up.	2	I was able to encourage patients to visit a dentist, but I do not follow up with them afterward. (G50)
	Difficult to provide for all patients.	2	It is difficult to implement for all patients with diabetes on outpatient visits. (D311)
	Lack of human resources.	1	Nurses understand the importance of providing patient education on oral health management, but we are facing a lack of human resources. (C307)

Themes, categories, and subcategories identified through qualitative content analysis of the semi-structured interviews are shown, along with the number of participants (n) who reported each subcategory and representative illustrative quotes.

Participant identifiers in parentheses (e.g., A426, G5) indicate the participant label (A–H, corresponding to Table 1) followed by the sequential quote number within that participant’s transcript.

Theme 1: Individual Learning Enhancement – The audiovisual material helped participants visualize patient approaches and oral assessments, improving their self-perceived ability to implement oral self-management education.

Theme 2: Professional Development and Reflection – Engagement with the material fostered participants’ self-reflection and professional growth, increasing motivation for oral health management practice and awareness of medical–dental collaboration.

Theme 3: Patient Care Insights – The learning experience enabled participants to gain a deeper understanding of patients’ perspectives and actual oral health conditions, identifying barriers such as patients’ low interest and limited awareness.

Theme 4: Implementation Considerations – Practical and organizational factors affected implementation; participants recognized both learning needs and strategies to reduce patients’ discomfort during oral observation.

#### Theme 1: Individual Learning Enhancement (Categories 1–2).

This theme represents nurses’ enhanced understanding and visualization of oral health management practices through audiovisual learning. Participants reported that the audiovisual format made it easier to understand how to approach patients and observe the oral cavity than text-based materials, offering clearer visual and auditory guidance for clinical practice. As one participant explained, “It was easier to understand how to approach patients because the information was not only presented in text, but also visually and audibly, with supporting images” (G5). This enhanced understanding was reflected in participants’ subsequent practice, as several nurses described delivering oral self-management education informed by the material.

#### Theme 2: Professional Development and Reflection (Categories 3–5).

This theme captures nurses’ professional growth and self-reflection prompted by exposure to the audiovisual material. Participants described increased motivation to practice oral health management and engaged in meaningful reflection on their prior clinical practice. As one nurse reflected, “I realized that I had not been practicing oral health management, even though it was necessary” (B176). The material also prompted reflection on the current state of medical-dental collaboration, revealing limited engagement with dental professionals in routine practice.

#### Theme 3: Patient Care Insights (Categories 6–7).

This theme describes how the learning experience deepened nurses’ awareness of patient perspectives and actual oral health conditions. Through applying oral health management practices, participants gained new insights into patients’ attitudes toward oral health, often encountering muted or disengaged responses: “Whatever I said, their reaction was rather muted—just ‘hmm,’ not much of a response” (A337). Participants also observed a range of oral conditions, from severe neglect to adequate self-care, and commonly identified barriers including patients’ low interest in oral health, discomfort with oral cavity observation, and limited awareness of the relationship between diabetes and periodontal disease.

#### Theme 4: Implementation Considerations (Categories 8–11).

This theme highlights the practical and organizational factors influencing the implementation of oral health management in nursing practice. Participants identified the scope of oral health management that nurses can feasibly perform in the diabetes outpatient setting, along with ongoing learning needs, strategies to facilitate implementation, and organizational barriers. Physical and organizational constraints were frequently cited, as one participant noted: “If we had a diabetes care room with a water supply and space for toothbrushing, we could observe [patients’ oral cavity]. However, the lack of such facilities makes it more difficult” (G67). All participants also emphasized the importance of reducing patients’ feelings of shame associated with oral cavity examination.

### Discussion

Themes 1 and 2 represent direct learning effects associated with the use of the audiovisual material, whereas Themes 3 and 4 reflect broader reflective and contextual insights that emerged as nurses engaged with the material in their practice. This discussion first addresses the learning-related outcomes directly linked to the audiovisual material, followed by consideration of the reflective and contextual insights that may inform future implementation and educational practice.

#### Impact on learning.

CDNEs can play a vital role in the oral health management of outpatients with diabetes [13]. This study explored how audiovisual material designed to support nurses’ patient education influenced CDNEs’ learning and educational practice in diabetes care. After using the audiovisual material, participants reported enhanced understanding and confidence in providing oral health education. This finding is consistent with a previous study of nursing home nurses, in which an educational video on standardized nursing language similarly improved nurses’ knowledge and professional confidence [24], supporting the potential value of audiovisual materials as an effective continuing education tool for nurses in clinical practice. The findings indicate that the material was perceived to have a positive influence on nurses’ learning, including enhancing individual learning, promoting professional development and reflection, and providing insights into patient care. For example, participants reported that watching the videos helped them visualize how to approach patients, observe the oral cavity, and deliver education—experiences that were directly linked to engagement with the audiovisual material. These findings suggest that such materials may serve as a useful educational resource for nurses in delivering patient education on oral health management for patients with diabetes. This is consistent with a systematic review reporting that oral health promotion interventions delivered by non-dental health care professionals, primarily nurses, improved both clinical outcomes (e.g., probing depth, attachment loss, plaque index) and non-clinical outcomes (e.g., knowledge, attitudes, and behaviors) among people with type 2 diabetes [25].

Enhancement of individual learning was identified as an effect of the audiovisual material. All participants reported that the material enhanced their understanding of how to approach patients, observe the oral cavity, identify oral symptoms, and provide patient education in practice. Participants particularly emphasized that the audiovisual format offered superior visualization compared to text-based materials. Two participants who had prior experience with the existing guide [14] noted that combining the videos with the guide appeared to deepen their understanding. This material was developed as a supplementary audiovisual resource to support visual learning for practical implementation in clinical settings, complementing the existing guide. The results suggest that these intended functions were largely achieved from the participants’ perspectives.

The audiovisual format appeared to facilitate the translation of knowledge into clinical practice. By demonstrating practical techniques visually, the material may have helped bridge the gap between theoretical understanding and hands-on patient care skills, addressing a common challenge in clinical education.

#### Professional reflection.

Participants reported that engaging with the audiovisual material prompted reflection on their current clinical practices. This finding aligns with a recent report on educational tools [15], in which visual learning materials were suggested to improve nurses’ comprehension of clinical procedures, facilitate the translation of knowledge into practice, and promote self-reflection on professional performance. Similarly, in our study, participants attributed their improved visualization and application of oral health management techniques primarily to their engagement with the audiovisual material. Moreover, participants reflected on the current state of medical-dental collaboration for patients with diabetes, revealing limited engagement with dental professionals in routine practice—a reflection stimulated by reviewing the audiovisual material.

A previous review reported a significant gap in medical-dental collaboration: although approximately 70% of dental hygienists recognized the need for such collaboration in managing patients with diabetes, only about 30% were actively engaged in it [26]. This gap reflects a longstanding divide between the medical and dental professions, in which dental health has historically been treated as separate from general health despite their close interrelation [27].

Although collaboration between medical and dental professionals is essential for maintaining oral health in patients with diabetes, such cooperation remains underdeveloped. In Japan, the 2024 revision of medical service fees by the Ministry of Health, Labour and Welfare introduced a reimbursement incentive for periodontal maintenance therapy targeting patients with diabetes at high risk of periodontal disease progression, which requires the patient’s physician to provide relevant medical information to the dental practitioner in writing [28]. This policy formalizes a documentation-based mechanism for sharing patients’ diabetes status between medical and dental care providers, rather than relying solely on informal encouragement to seek dental consultation. This policy aims to promote more effective disease management and prevent progression to serious complications in response to the growing prevalence of chronic diseases such as diabetes. The Ministry also highlighted the need for improved collaboration among medical departments, dispensing pharmacies, and dental care services. Therefore, further strategies to enhance medical-dental collaboration in Japan should be developed, including practical collaboration models that integrate medical departments, pharmacies, and dental services into a cohesive care framework. In this policy context, the audiovisual material may serve as a practical educational tool enabling nurses to respond to these expectations by supporting oral health education within medical settings.

#### Patient insights.

After using the audiovisual material in patient education, the majority of participants reported gaining new insights into patient perspectives on oral health management. Participants noted that patients with diabetes demonstrated low levels of interest in oral health, and many were unaware of the relationship between diabetes and periodontal disease. Regarding actual oral conditions, half of the nurses observed very poor oral hygiene and tooth loss that interfered with eating. These observations were described in relation to applying knowledge and perspectives gained from the audiovisual material. Creating a supportive environment for patient education, as highlighted by participants, was linked to the strategies demonstrated in the material.

Although some patients were aware of their oral health issues, they often did not perceive them as particularly concerning and delayed seeking dental care until symptoms became severe. These observations align with existing literature. A systematic review reported that fewer than 50% of patients with diabetes were aware of the bidirectional relationship between diabetes and periodontal disease [29], suggesting that patients may not receive adequate information about this relationship from healthcare providers. Another systematic review demonstrated that oral health behaviors can be improved when medical professionals provide patients with information or refer them to dental care [30]. These findings underscore the importance of enhancing patient education on oral health within medical settings and the need for continuous professional development to help medical professionals maintain current knowledge in this area.

Participants recognized the need for sensitivity when discussing oral health with patients. The CDNEs in this study reported that their patients expressed feelings of shame and discomfort when being asked questions about oral health or having their oral cavity observed. These observations align with previous research indicating that patients may experience embarrassment about their dental condition during oral examinations [15]. Although some participants reported that certain patients showed no resistance to oral health inquiries or examinations, healthcare providers should remain attentive to patients’ emotional responses during such assessments. Creating a supportive, non-judgmental environment may facilitate more effective oral health discussions and improve patient engagement in preventive care.

Interestingly, some participants observed that patients who neglected oral care also tended to neglect foot care, suggesting these behaviors might reflect broader attitudes toward self-care and health management. Participants speculated that a patient’s oral condition might serve as a predictor of foot condition and overall self-care engagement. However, this association has not been systematically investigated. Future research should explore whether oral health status can serve as an indicator of general self-care behaviors in patients with diabetes, which could inform more comprehensive care approaches and early intervention strategies.

#### CDNEs-reported education implementation challenges.

Participants identified that the scope of oral health management nurses can perform in diabetes outpatient departments includes oral health instruction and oral cavity observation. Educational support for nurses should be further developed based on these clinical realities. Many of these insights emerged when participants applied the audiovisual material in their clinical practice, which helped them recognize practical and organizational factors influencing implementation. To facilitate future implementation, nurses requested additional resources, including more images depicting diverse oral conditions and examples of oral health management support from other facilities. These requests and reflections were based on their experiences using the audiovisual material in practice. Some participants also expressed the need for practical consolidation of audiovisual learning through hands-on application. These findings underscore the importance of supporting nurses in establishing patient education methods by reinforcing audiovisual learning with practical experience. Furthermore, sharing information from facilities already engaged in oral health management practices may promote broader adoption of these approaches. Establishing networks that connect healthcare professionals with shared interests in oral health management could further enhance dissemination and implementation.

As strategies to facilitate patient acceptance, all participants emphasized the necessity of reducing patients’ feelings of shame when their oral cavity is observed, and most identified the need for a trigger to initiate oral cavity observation. These findings are consistent with recent literature indicating that the state of a person’s oral cavity and its visibility to others can be a significant source of shame, which may act as a barrier to seeking or engaging in oral health care, underscoring the need for non-judgmental, shame-sensitive approaches when patients’ oral cavities are observed or discussed [31]. These findings highlight the need for measures such as providing private rooms to ensure privacy and informing patients in advance of oral health education.

Several nurses reported that patients’ oral problems became apparent during discussions about dietary habits. Given that oral problems, including periodontal disease, can lead to tooth loss, impaired mastication, and nutritional deficiencies [10], conversations about dietary habits may serve as an effective trigger for initiating patient education on oral health. Some nurses proposed innovative educational approaches, including audiovisual materials for patients, telenursing, and the use of oral cavity models for patient education. Regarding telenursing specifically, a recent umbrella review demonstrated that nurse-led remote care can achieve outcomes comparable to, or in some cases better than, traditional face-to-face care in diabetes management [32], suggesting that a telenursing approach to oral health education may similarly hold promise, although its specific application to oral cavity observation has not yet been reported. As these approaches have not yet been widely reported in the literature, their introduction into clinical practice warrants further consideration and evaluation.

Nurses in this study raised concerns about the absence of additional medical reimbursement for oral health management. The lack of a designated medical fee for oral health management of patients with diabetes has been identified as a barrier to providing such care in medical settings [33]. Nurses who recognized the importance of oral health management and expressed willingness to engage appeared to view the establishment of supportive physical and organizational environments as more critical than addressing psychological factors alone. Organizational support is closely tied to the availability of medical reimbursement, and several implementation challenges reported in this study—including limited time, lack of interest from other staff, and insufficient human resources—may be addressed through stronger organizational commitment. However, evidence in this field remains limited. Accumulating evidence of intervention effectiveness is essential to establish medical fee structures for oral health management of patients with diabetes in medical settings. Such evidence-based support would provide the foundation for sustainable implementation of oral health programs within diabetes care.

The study results suggest that participants perceived the audiovisual material to enhance nurses’ individual learning, promote professional development and reflection, and provide new insights into patient perspectives. The development and implementation of this material are significant, as they assist nurses in practicing oral health management and contribute to promoting oral health education for patients. Nurses who had previously used the existing guide [14] reported that combining the videos with the guide helped deepen their understanding. It is recommended that future practice integrate both the guide and audiovisual materials into a comprehensive educational program. This multimodal approach may optimize learning outcomes and facilitate the translation of knowledge into clinical practice.

### Strengths and limitations

This study has several strengths. First, it provides qualitative insights into how audiovisual materials can support nurses’ learning and patient education practices in oral health management within diabetes care—an underexplored area. Second, the use of audiovisual materials combined with qualitative content analysis allowed for an in-depth understanding of nurses’ perceptions and learning processes, which may inform future educational interventions.

Several limitations should be acknowledged. First, only eight of 122 CDNEs initially recruited agreed to participate. This low response rate may have been influenced by the inability to send reminder invitations and the absence of participant compensation. As this study employed a qualitative approach, sample size was guided by the depth and richness of the data obtained rather than by statistical power considerations. Nonetheless, the relatively small number of participants may have introduced selection bias, as those who agreed to participate likely had greater interest in oral health management, and the transferability of these findings to other settings should be considered with appropriate caution. Additionally, time constraints may have influenced participation despite scheduling adjustments. Although recurring themes emerged across interviews, the limited number of participants does not permit a claim of data saturation in the strict methodological sense, and additional interviews may have yielded new concepts. The findings should be interpreted as exploratory. Future studies should employ strategies to improve recruitment, such as multiple contact attempts, flexible participation formats, and appropriate incentives.

Second, the cross-sectional design limited our ability to evaluate long-term impacts. Third, we did not confirm how participants actually used the audiovisual material during patient education, relying instead on self-reported numbers of patients educated. Fourth, this study did not assess patient-centered outcomes such as improvements in oral health status or glycemic control. Fifth, member checking with participants was not performed in this study; therefore, the interpretations presented reflect the researchers’ analysis of the interview data rather than participant-confirmed accounts, which may have limited the credibility and confirmability of the findings. Sixth, at the time of this study, oral cavity observation and assessment were not formally defined within the certified scope of practice for CDNEs, and the material’s approach to linking nurses’ findings with dental professionals was limited to encouraging patients to seek dental consultation, without a structured referral system. Future development of the material, and of institutional practice more broadly, should consider establishing clearer linkages between nursing observation and dental referral pathways, as well as clarifying the scope of nursing practice in oral health assessment. The observed effects on nurses provide a foundation for future investigations evaluating the material’s impact on patient health. Future research should:

1. expand evaluation to include patient-centered outcomes in order to provide a more comprehensive assessment of the material’s effectiveness,
2. conduct a large-scale mixed-methods study combining quantitative evaluation of the educational material’s effectiveness with qualitative exploration of implementation experiences,
3. perform longitudinal follow-up studies to assess the sustainability of changes in practice, and
4. investigate organizational factors that support or impede the adoption of oral health management practices in diabetes care settings.

## Conclusions

The study suggests the potential usefulness of audiovisual material in supporting nurses in delivering patient education on oral health management in diabetes care. The material was perceived by participants as useful and appeared to promote individual learning, professional development and reflection, and to provide insights into patient care. In addition to these perceived learning-related benefits, the study identified several implementation-related considerations and contextual insights that emerged as nurses reflected on applying the material in clinical practice. Given the small sample size (n = 8) and the exploratory nature of this study, these findings should be interpreted with caution. While the audiovisual material showed promise in supporting nurses’ self-perceived learning and educational practices, further research with larger and more diverse samples is needed to establish its effectiveness more definitively.

Future studies should expand evaluation to include patient-centered outcomes, confirm how nurses actually use the audiovisual material in practice, and conduct larger-scale mixed-methods research to establish stronger evidence for the material’s effectiveness and practical utility. Additionally, since only two participants had prior experience with the existing evidence-based guide, future research should systematically examine the combined use of both resources to determine whether their integration provides added value beyond using either resource alone.

These findings highlight the potential importance of integrating oral health management into diabetes care and education. Based on this exploratory study, healthcare institutions and nursing educators may wish to consider the potential value of audiovisual educational materials as a supplementary resource within training programs, with the aim of enhancing nurses’ competence in delivering oral health education to patients with diabetes. CDNEs may find such materials useful for strengthening their knowledge and skills in oral health assessment and patient education, while also fostering reflection on current clinical practices and the need for medical-dental collaboration. Given the small sample size and exploratory nature of this study, these implications should be regarded as preliminary, and further research with larger, more diverse samples is needed before the integration of such materials into training programs can be more firmly recommended.

## Supporting information

S1 File STROBE Statement—Checklist of items that should be included in reports of *cross-sectional studies.* (DOCX)
